# North-American Medico-Chirurgical Review

**Published:** 1861-08

**Authors:** 


In addition to its strictly professional matter, the *Medico-Chirugical* presents the claims of the author of *Don Quixote* to the title of *Medicinæ Doctor*, as advanced by late French and Spanish writers; some of whom not only do not hesitate to assign Cervantes' master-piece a high rank as a treatise on insanity, but for Cervantes, himself, a priority to Pinel in the moral treatment of this disease, and to Broussais in recognizing the intimate connections between the digestive apparatus and its function and the general health, physical and mental. Despite the reviewer, however, we doubt if Sydenham recognized this claim when he recommended the *Quixote* as the best book for a medical student to read.

The editorial department of the *Medico-Chirugical* is a field of the poetry and romance of our profession, most fascinating to the imaginative and enthusiastic student of its various phases.

